# Mitochondrial genomes of two Australian fishflies with an evolutionary timescale of Chauliodinae

**DOI:** 10.1038/s41598-017-04799-y

**Published:** 2017-06-30

**Authors:** Fan Yang, Yunlan Jiang, Ding Yang, Xingyue Liu

**Affiliations:** Department of Entomology, China Agricultural University, Beijing, 100193 China

## Abstract

Fishflies (Corydalidae: Chauliodinae) with a total of ca. 130 extant species are one of the major groups of the holometabolous insect order Megaloptera. As a group which originated during the Mesozoic, the phylogeny and historical biogeography of fishflies are of high interest. The previous hypothesis on the evolutionary history of fishflies was based primarily on morphological data. To further test the existing phylogenetic relationships and to understand the divergence pattern of fishflies, we conducted a molecule-based study. We determined the complete mitochondrial (mt) genomes of two Australian fishfly species, *Archichauliodes deceptor* Kimmins, 1954 and *Protochauliodes biconicus* Kimmins, 1954, both members of a major subgroup of Chauliodinae with high phylogenetic significance. A phylogenomic analysis was carried out based on 13 mt protein coding genes (PCGs) and two rRNAs genes from the megalopteran species with determined mt genomes. Both maximum likelihood and Bayesian inference analyses recovered the *Dysmicohermes* clade as the sister group of the *Archichauliodes* clade + the *Protochauliodes* clade, which is consistent with the previous morphology-based hypothesis. The divergence time estimation suggested that the divergence among the three major subgroups of fishflies occurred during the Late Jurassic and Early Cretaceous when the supercontinent Pangaea was undergoing sequential breakup.

## Introduction

The subfamily Chauliodinae, commonly known as fishflies, is one of the three major groups of the holometabolous order Megaloptera. It belongs to the family Corydalidae, which also includes the subfamily Corydalinae (dobsonflies) and which is regarded as the sister group to the family Sialidae. Compared to Corydalinae, adult fishflies can be distinguished by the absence of postocular plane on head, the reduced cross venation, the callus cerci present on ectoprocts, and the reduced male gonostylus 9^1, 2^. Fishfly larvae are easily recognized by the absence of ventral tufts and the presence of specialized spiracles on abdominal segment 8^3, 4^. Currently, over 130 species in 18 genera of extant fishflies are known worldwide^2^.

Fishflies live mainly in the subtropical or warm temperate regions, and they occur in all zoogeographical realms. However, they show a remarkably discontinuous distribution due to their absence in the western Palaearctic realm and most parts of the Afrotropical and Neotropical realms^4^. Fishflies are an archaic insect group and many of the extant species qualify as “living fossils”, since they originated no later than the Middle Jurassic based on the fossil evidence and display remarkably conservative, unchanged adult and larval morphology between Mesozoic fossil and modern species^4^. Hence, the phylogeny and historical biogeography of fishflies are of high interest and have been recently studied by Liu & Yang (2006), Liu *et al*. (2012, 2016) and Wang *et al*. (2012). The current phylogenetic framework of Chauliodinae subdivides the subfamily into three extant groups, i.e., the *Dysmicohermes* clade, the *Protochauliodes* clade and the *Archichauliodes* clade^4^. The *Dysmicohermes* clade comprises only two western Nearctic endemic genera *Dysmicohermes* and *Orohermes*. The *Protochauliodes* clade is composed of *Madachauliodes*, *Neohermes*, *Nothochauliodes*, *Protochauliodes* and *Taeniochauliodes*, many of which are distributed in the Southern Hemisphere except *Neohermes* and some species of *Protochauliodes* from North America. The *Archichauliodes* clade includes *Platychauliodes* from South Africa, *Archichauliodes* and *Apochauliodes* from Australia, New Zealand and Chile, and all Asian fishfly genera. The modern fauna of fishflies is thought to be formed by the divergence associated with the sequential breakup and drifting of Gondwana^4^.

However, despite the divergence between Chauliodinae and Corydalinae, which was estimated by a molecular approach^5^ to have occurred during the Early Jurassic (~186 MA), all previous hypotheses on phylogeny and historical biogeography of Chauliodinae were proposed without the use of molecular data.

Here we determined and describe the complete mitochondrial (mt) genomes of two fishfly species, namely *Archichauliodes deceptor* Kimmins, 1954 and *Protochauliodes biconicus* Kimmins, 1954. Both are endemic to Australia and are the first fishfly species from the Southern Hemisphere for which the mt genome has been determined. The genome organization, protein-coding genes, transfer RNAs, ribosomal RNAs and the control region were analyzed. A phylogenomic analysis was performed with known mt genome data of Megaloptera to infer the phylogenetic positions of *Archichauliodes* and *Protochauliodes* and to test the previous phylogeny of Chauliodinae based on morphological data. Furthermore, the evolutionary pattern of the three major subgroups of Chauliodinae was reconstructed based on divergence time estimation. The results corroborated the relationships of the three major subgroups of fishflies that was based on morphological data. Additionally, the first molecule-based timescale on the early divergence of fishflies is presented. Lastly, the historical biogeography of fishflies is discussed in light of the new evidence from the molecular data.

## Results

### Genome organization and structure

**Figure 1:**
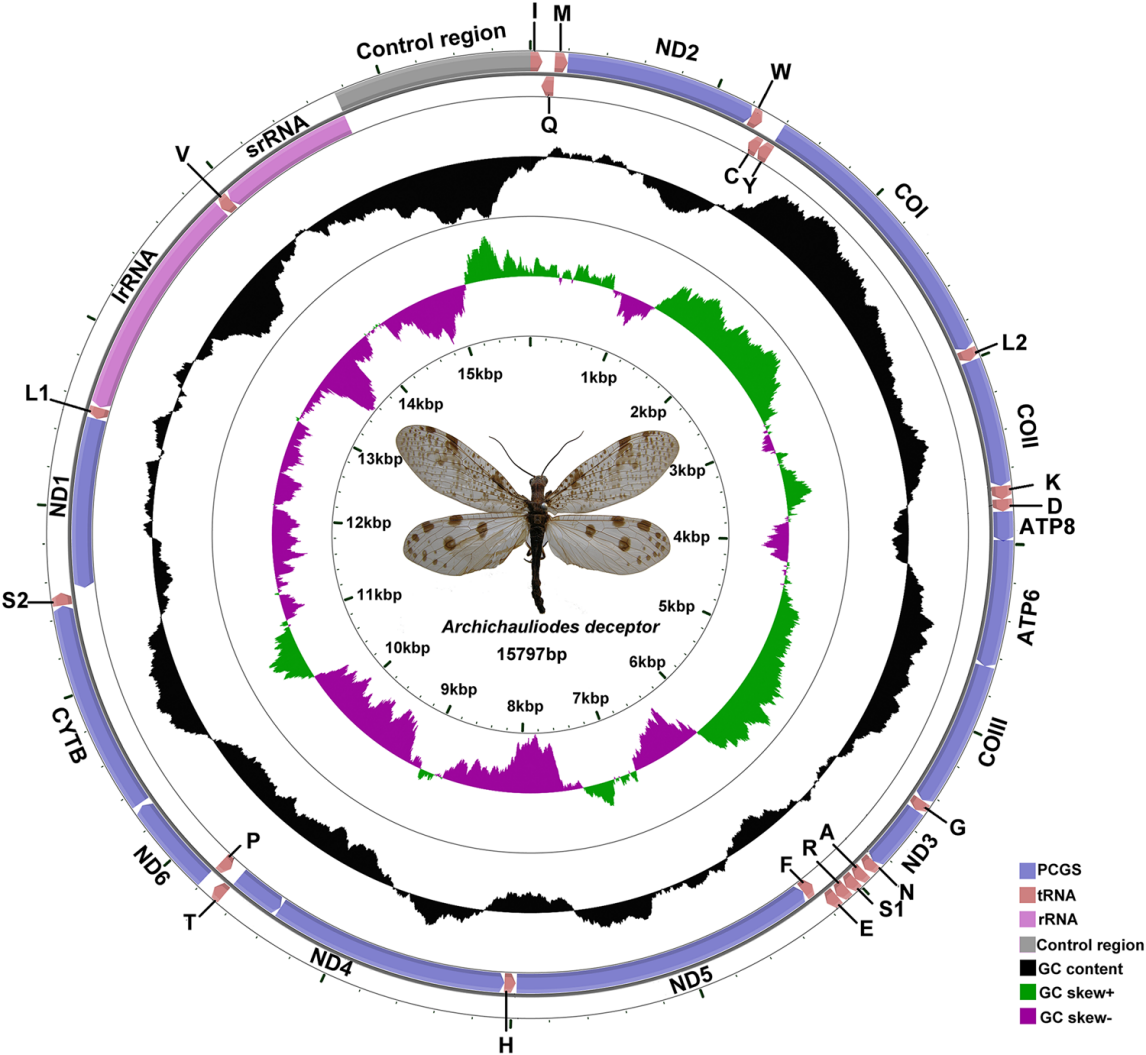
Mitochondrial map of *Archichauliodes deceptor*. Circular maps were drawn with CGView^25^. The arrows indicate the orientation of gene transcription. The tRNAs are denoted by the color blocks and are labelled according to the IUPACIUB single-letter amino acid codes (L1: UUR; L2: CNU; S1: AGN; S2: UCN). The GC content was plotted using a black sliding window, as the deviation from the average GC content of the entire sequence. GC-skew was plotted as the deviation from the average GC-skew of the entire sequence. The inner cycle indicates the location of the genes in the mt genome.

**Figure 2:**
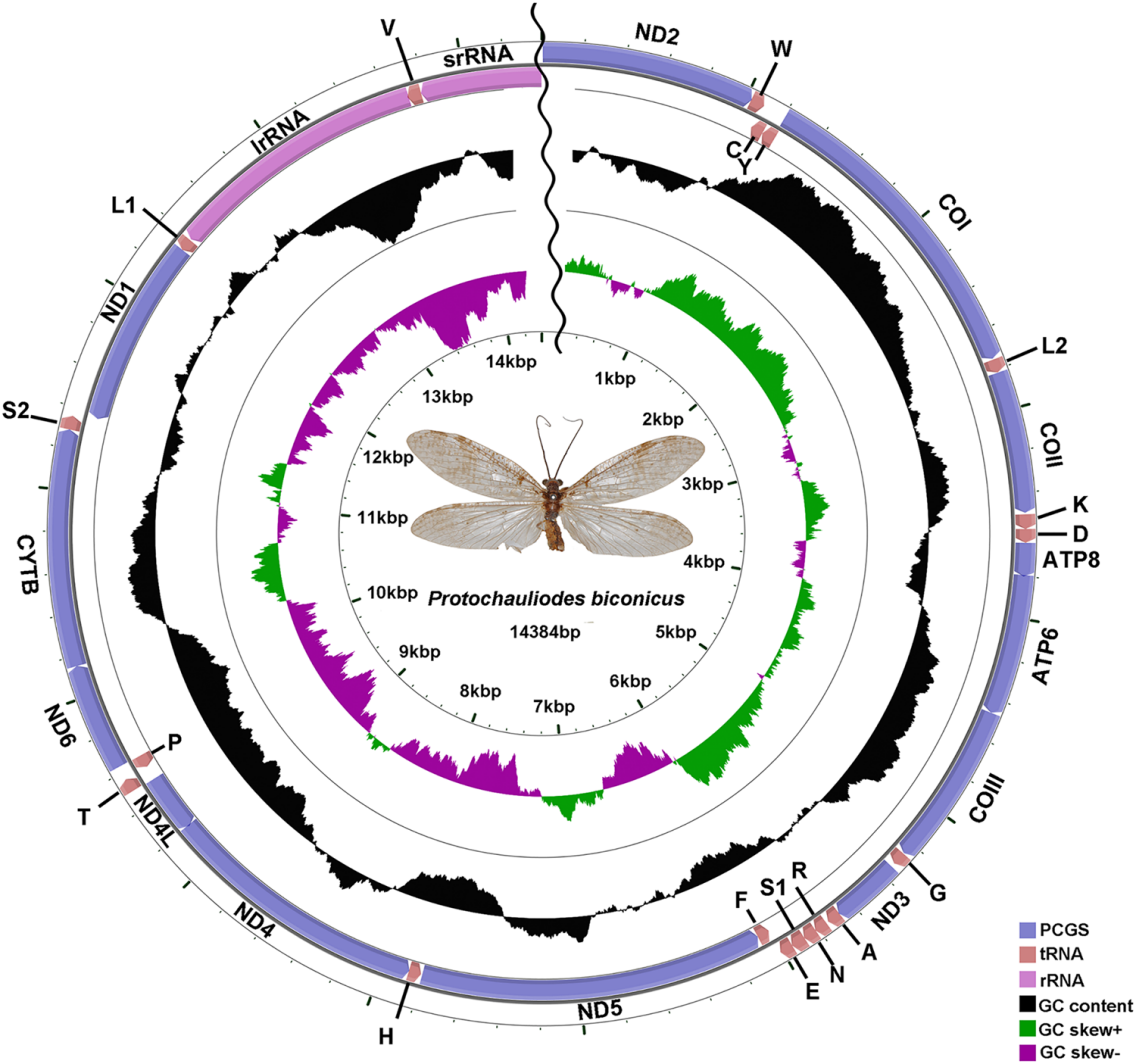
Mitochondrial map of *Protochauliodes biconicus*. Circular maps were drawn with CGView^25^. The arrows indicate the orientation of gene transcription. The tRNAs are denoted by the color blocks and are labelled according to the IUPACIUB single-letter amino acid codes (L1: UUR; L2: CNU; S1: AGN; S2: UCN). The GC content was plotted using a black sliding window, as the deviation from the average GC content of the entire sequence. GC-skew was plotted as the deviation from the average GC-skew of the entire sequence. The inner cycle indicates the location of the genes in the mt genome.

**Figure 3:**
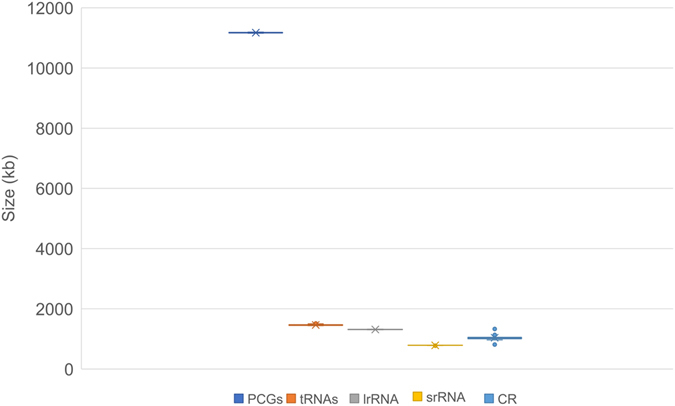
The size of PCGs, *lrRNA*, *srRNA* and CR, respectively, among sequenced Megaloptera mt genomes.

The complete mt genome of *A*. *deceptor* is a typical circular, double-strand molecule of 15,797 bp in length (GenBank accession number: KU925864; Fig. 1, Table 1), which is relatively small in size compared to the mt genomes of Megaloptera known thus far, with length ranging from 15,687 bp (*Corydalus cornutus*, Corydalidae, NC_011226) to 16,271 bp (*Dysmicohermes ingens*, Corydalidae, NC_16271). The mt genome contains 37 genes, including 22 tRNAs, 13 PCGs, two rRNAs and a control region. The sequenced part of the *P*. *biconicus* mt genome is 14,384 bp in length and contains 34 genes with 19 tRNAs, 13 PCGs, *lrRNA* and partial *srRNA* (Fig. 2, Table 2). Three tRNAs (i.e., *tRNA* ^*Ile*^, *tRNA* ^*Gln*^, *tRNA* ^*Met*^) and the control region failed to be amplified probably due to high variation and complex secondary structures of this part. In the Megaloptera mt genomes, variations in the length of PCGs, tRNAs, *lrRNA* and *srRNA* are inconspicuous except for the length of the control region (see Fig. 3; Table S1).

**Table 1:** Organization of the *Archichauliodes deceptor* mt genome.

Gene	Direction	Location	Size (bp)	IGN*	Anticodon	Codon		AT%
						Start	Stop	
*tRNA* ^*Ile*^	F	1–64	64	0	GAT			68.8
*tRNA* ^*Gln*^	R	62–130	69	−3	TTG			79.7
*tRNA* ^*Met*^	F	131–199	69	0	CAT			71
*ND2*	F	200–1222	1023	0		ATT	TAA	79.5
*tRNA* ^*Trp*^	F	1221–1285	65	−2	TCA			80
*tRNA* ^*Cys*^	R	1277–1341	63	−9	GCA			81
*tRNA* ^*Tyr*^	R	1341–1411	66	−1	GTA			74.2
*COI*	F	1398–2939	1542	−4		ATT	TAA	69.2
*tRNA* ^*Leu*(*UUR*)^	F	2941–3004	64	1	TAA			76.6
*COII*	F	3007–3691	685	2		ATG	T−	73.1
*tRNA* ^*Lys*^	F	3692–3762	71	0	CTT			74.6
*tRNA* ^*Asp*^	F	3762–3827	66	−1	GTC			83.3
*ATP8*	F	3828–3986	159	0		ATC	TAA	82.4
*ATP6*	F	3980–4657	678	−7		ATG	TAA	74.5
*COIII*	F	4657–5445	789	−1		ATG	TAA	71.7
*tRNA* ^*Gly*^	F	5448–5509	62	2	TCC			82.2
*ND3*	F	5510–5863	354	0		ATT	TAG	78.8
*tRNA* ^*Ala*^	F	5862–5924	63	−2	TGC			71.4
*tRNA* ^*Arg*^	F	5935–5997	64	10	TCG			71.9
*tRNA* ^*Asn*^	F	5997–6062	66	−1	GTT			80.3
*tRNA* ^*Ser*(*AGN*)^	F	6063–6129	67	0	GCT			73.2
*tRNA* ^*Glu*^	F	6130–6195	66	0	TTC			89.4
*tRNA* ^*Phe*^	R	6194–6258	65	−2	GAA			78.5
*ND5*	R	6259–7975	1722	0		ATA	T−	77.5
tRNA^His^	R	7982–8044	63	6	GTG			81
*ND4*	R	8045–9383	1339	0		ATG	T−	78.9
*ND4L*	R	9377–9670	294	−7		ATG	TAA	79.6
*tRNA* ^*Thr*^	F	9673–9737	65	2	TGT			81.5
*tRNA* ^*Pro*^	R	9738–9803	66	0	TGG			81.8
*ND6*	F	9809–10318	510	5		ATT	TAA	83
*CYTB*	F	10335–11471	1137	16		ATG	TAA	73.5
*tRNA* ^*Ser*(*UCN*)^	F	11474–11540	67	2	TGA			86.6
*ND1*	R	11553–12503	951	12		TTG	TAA	76.8
*tRNA* ^*Leu*(*CUN*)^	R	12505–12568	64	1	TAG			81.3
*lrRNA*	R	12572–13889	1318	3				82
*tRNA* ^*Val*^	R	13891–13961	71	1	TAC			77.5
*srRNA*	R	13959–14748	790	−3				79.4
Control region	—	14749–15798	1050	0				86.8

IGN: Intergenic nucleotide, minus sign indicates overlapping between genes. tRNA^X^: where X is the abbreviation of the corresponding amino acid.

**Table 2:** Organization of the *Protochauliodes biconicus* mt genome.

Gene	Direction	Location	Size (bp)	IGN*	Anticodon	Codon		AT%
						Start	Stop	
*ND2*	F	5–1027	1023	0		ATT	TAA	79.3
*tRNA* ^*Trp*^	F	1026–1092	67	−2	TCA			76.1
*tRNA* ^*Cys*^	R	1085–1147	63	−8	GCA			73.0
*tRNA* ^*Tyr*^	R	1148–1213	66	0	GTA			66.7
*COI*	F	1206–2745	1540	−8		ATT	T−	69.1
*tRNA* ^*Leu*(*UUR*)^	F	2751–2815	65	5	TAA			73.9
*COII*	F	2817–3498	682	5		ATG	T−	74.5
*tRNA* ^*Lys*^	F	3506–3576	71	7	CTT			69.0
*tRNA* ^*Asp*^	F	3576–3641	66	−1	GTC			80.3
*ATP8*	F	3642–3800	159	0		ATT	TAA	81.8
*ATP6*	F	3794–4469	676	−7		ATG	T−	75.4
*COIII*	F	4470–5258	789	0		ATG	TAA	70.7
*tRNA* ^*Gly*^	F	5262–5325	64	3	TCC			79.7
*ND3*	F	5345–5680	336	19		ATA	TAG	76.5
*tRNA* ^*Ala*^	F	5679–5743	65	−2	TGC			76.9
*tRNA* ^*Arg*^	F	5755–5817	63	11	TCG			74.3
*tRNA* ^*Asn*^	F	5817–5881	65	−1	GTT			78.5
*tRNA* ^*Ser*(*AGN*)^	F	5881–5949	69	−1	GCT			75.4
*tRNA* ^*Glu*^	F	5949–6012	64	−1	TTC			89.1
*tRNA* ^*Phe*^	R	6011–6072	62	−2	GAA			77.4
*ND5*	R	6073–7797	1725	0		ATA	TAA	78.1
*tRNA* ^*His*^	R	7804–7866	63	6	GTG			84.2
*ND4*	R	7865–9206	1342	−2		ATA	T−	78.8
*ND4L*	R	9200–9494	300	−7		ATA	TAA	83.4
*tRNA* ^*Thr*^	F	9500–9564	65	5	TGT			83.1
*tRNA* ^*Pro*^	R	9565–9629	65	0	TGG			80.0
*ND6*	F	9632–10153	522	2		ATT	TAA	85.2
*CYTB*	F	10153–11289	1137	−1		ATG	TAA	73.9
*tRNA* ^*Ser*(*UCN*)^	F	11289–11355	67	−1	TGA			82.1
*ND1*	R	11371–12325	955	15		ATT	T−	73.8
*tRNA* ^*Leu*(*CUN*)^	R	12325–12387	63	−1	TAG			79.4
*lrRNA*	R	12394–13711	1318	6				81.7
*tRNA* ^*Val*^	R	13706–13776	71	−6	TAC			70.4
*srRNA*	R	13776–14384	610	−1				80.1

IGN: Intergenic nucleotide, minus sign indicates overlapping between genes. tRNA^X^: where X is the abbreviation of the corresponding amino acid.

The gene order is in accordance with the gene order of *Drosophila yakuba* ^6^, and no gene rearrangement was found. The published mt genomes of all 12 species of Megaloptera exhibit a highly conserved gene order^7^. However, the gene order of some reported Neuroptera mt genomes differs slightly from the conserved gene order in the translocation of *tRNA* ^*Cys*^, which is located upstream of *tRNA* ^*Trp*^ but not at its traditional downstream location of *tRNA* ^*Trp*^. Among all 37 genes in the *A*. *deceptor* mt genome, 14 genes (4 PCGs, 2 rRNAs and 8 tRNAs) are encoded on the minority strand (N-strand), and 23 genes (9 PCGs and 14 tRNAs) are on the majority strand (J-strand). Among 33 genes in the partial *P*. *biconicus* mt genome, 12 genes (4 PCGs, 1 rRNA and 7 tRNAs) are encoded on the minority strand (N-strand), and 21 genes (9 PCGs and 12 tRNAs) are on the majority strand (J-strand). Gene overlaps were found at 12 and 16 gene junctions in the mt genomes of *A*. *deceptor* and *P*. *biconicus*, respectively. Furthermore, *ATP6* and *ATP8* overlap 7 nucleotides (i.e., “ATGATAA”), and this phenomenon is also reported in the mt genome of some related species (e.g., *Neochauliodes bowringi* (McLachlan), *Neochauliodes punctatolosus* Liu & Yang, *Protohermes concolorus* Yang & Yang). Similarly, *ND4L*-*ND4* also had a 7 bp overlap (i.e., “TTAACAT”), but the overlapped sequences between *ND4L*-*ND4* were not always the same in insect mt genomes, such as “TTAACAC” in *N*. *bowringi* and “ATGTTAA” in *N*. *punctatolosus*. In addition, there were 13 intergenic regions in the mt genome including 65 nucleotides and ranging from 1 to 16 bp in the *A*. *deceptor* mt genome. In the *P*. *biconicus* mt genome, 12 intergenic regions were found, including 96 nucleotides which ranged from 1 to 19 bp. Fifteen non-coding regions were found in the whole mt genome of *A*. *deceptor*, and the largest non-coding region had 1050 bp and was located between *srRNA* and *tRNA* ^*Ile*^ with the A + T content at 86.8%.

### Protein-coding genes

The overall A + T content of all 13 PCGs in the genomes of *A*. *deceptor* and *P*. *biconicus* was 75.8% and 76%, respectively. Twelve PCGs in the *A*. *deceptor* mt genome and all 13 PCGs in the *P*. *biconicus* mt genome use ATN as the start codon, the only exception refers to the *ND1*, which initiate with TTG in the *A*. *deceptor* mt genome. The stop codons mostly used are TAA, while in the mt genome of two species, *ND3* used TAG as the stop codon, and the other genes used an incomplete stop codon T.

### Transfer RNAs

**Figure 4:**
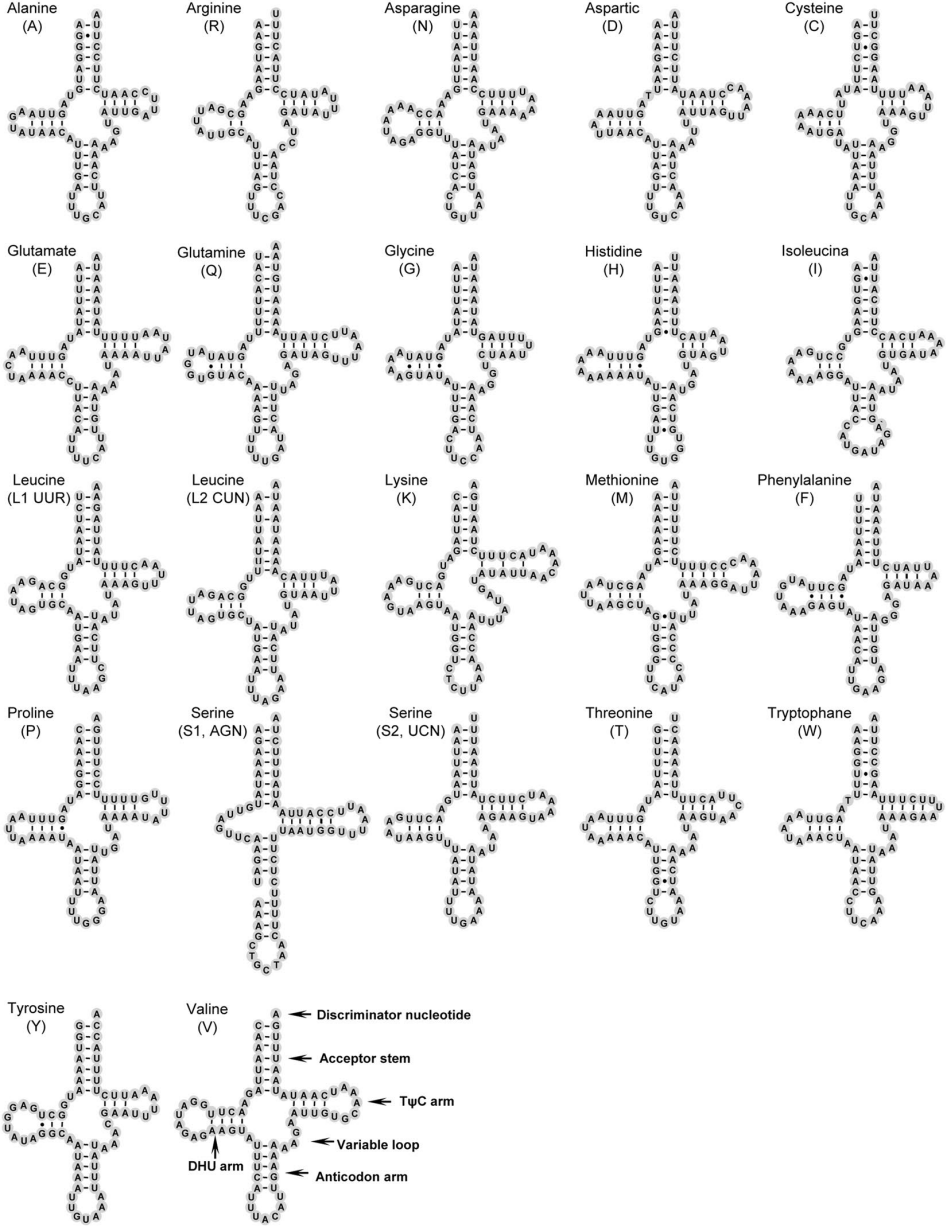
Inferred secondary structure of 22 tRNAs in the *Archichauliodes deceptor* mt genome. Most tRNAs are labeled with the abbreviations of their corresponding amino acids. Dash (−) indicates Watson-Crick bonds and dot (∙) indicates GU bonds.

**Figure 5:**
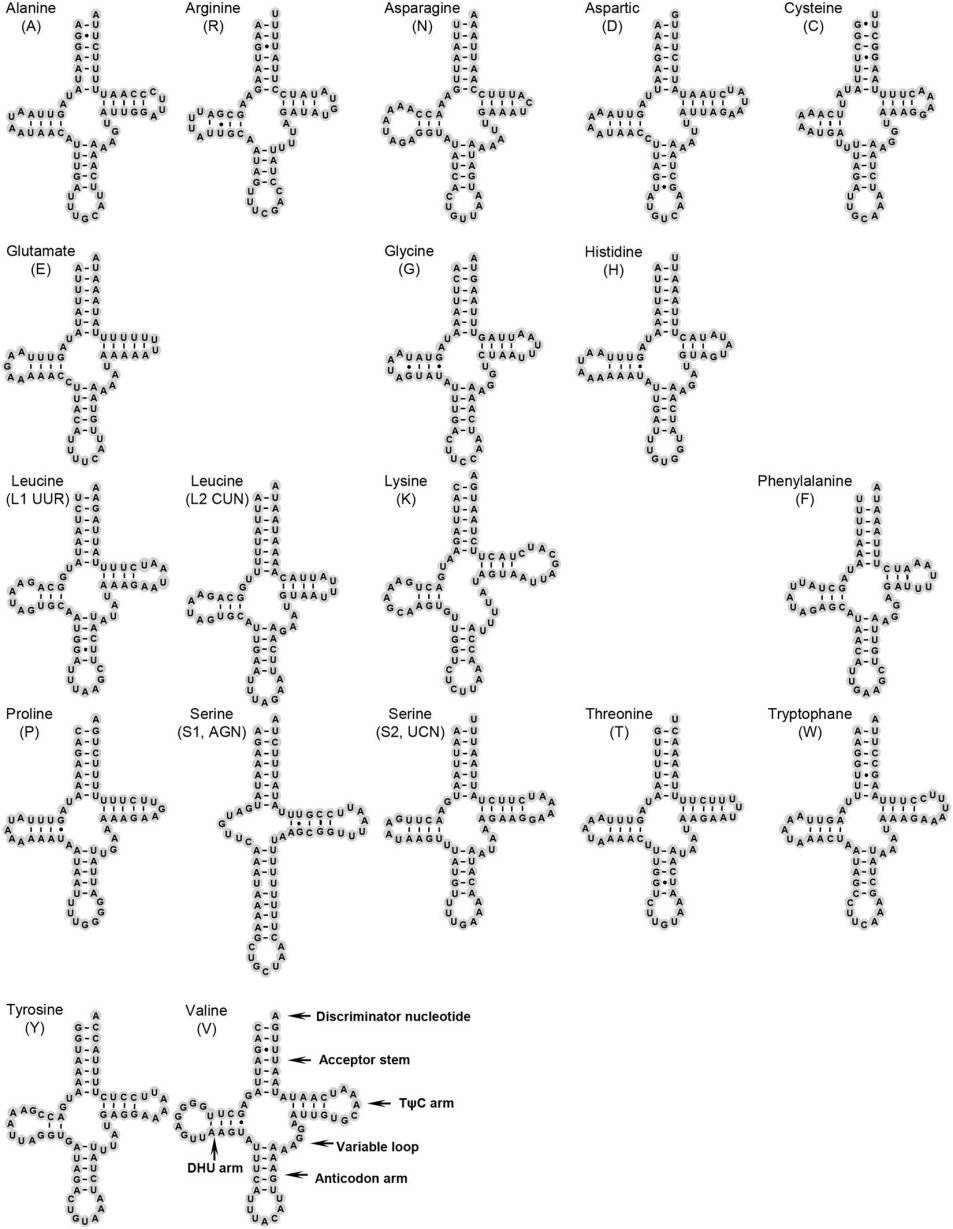
Inferred secondary structure of 19 tRNAs in the *Protochauliodes biconicus* mt genome. Most tRNAs are labeled with the abbreviations of their corresponding amino acids. Dash (−) indicates Watson-Crick bonds and dot (∙) indicates GU bonds.

According to the secondary structure and corresponding anticodon of tRNAs, we identified 22 tRNA genes in the mt genome of *A*. *deceptor*, ranging in size from 63 bp (*tRNA* ^*Gly*^) to 71 bp (*tRNA* ^*Lys*^, *tRNA* ^*Val*^). In the partial mt genome of *P*. *biconicus*, there were 19 tRNA genes ranging in size from 62 bp (*tRNA* ^*Phe*^) to 71 bp (*tRNA* ^*Lys*^, *tRNA* ^*Val*^). Fourteen tRNA genes in *A*. *deceptor* and 12 genes in *P*. *biconicus* are encoded on the J-strand, while the remaining tRNAs are encoded on the N-strand. Most tRNAs could be folded as typical clover-leaf structures except for *tRNA* ^*Ser*(*AGN*)^ due to lack of the DHU arm (Figs 4 and 5), and the AC arm of *tRNA* ^*Ser*(*AGN*)^ was a stem structure with an independent nucleotide in the middle. This phenomenon is common in sequenced Neuropterida mt genomes. The DHU and TψC stems are more variable and range in size from 3 bp to 10 bp.

Based on the secondary structure, the amount of mismatched base pairs in tRNAs of *A*. *deceptor* and *P*. *biconicus* was 21 and 19, respectively, this includes G-U pairs, A-A mismatches, U-U mismatches and U-C mismatch. These mismatches were mostly found in the 3′ end of acceptor stem and DHU arm.

### Ribosomal RNAs

The *lrRNA* was assumed to fill up the blanks between *tRNA* ^*Leu*(*CUN*)^ and *tRNA* ^*Val*^, while the *srRNA* was flanked by *tRNA* ^*Val*^ and the control region. In *A*. *deceptor* the length of *lrRNA* and *srRNA* was determined to be 1319 bp and 786 bp with A + T content as 82% and 79.4%, respectively. The *lrRNA* of *P*. *biconicus* is 1318 bp in length, while the size of *srRNA* we sequenced is 610 bp.

**Figure 6:**
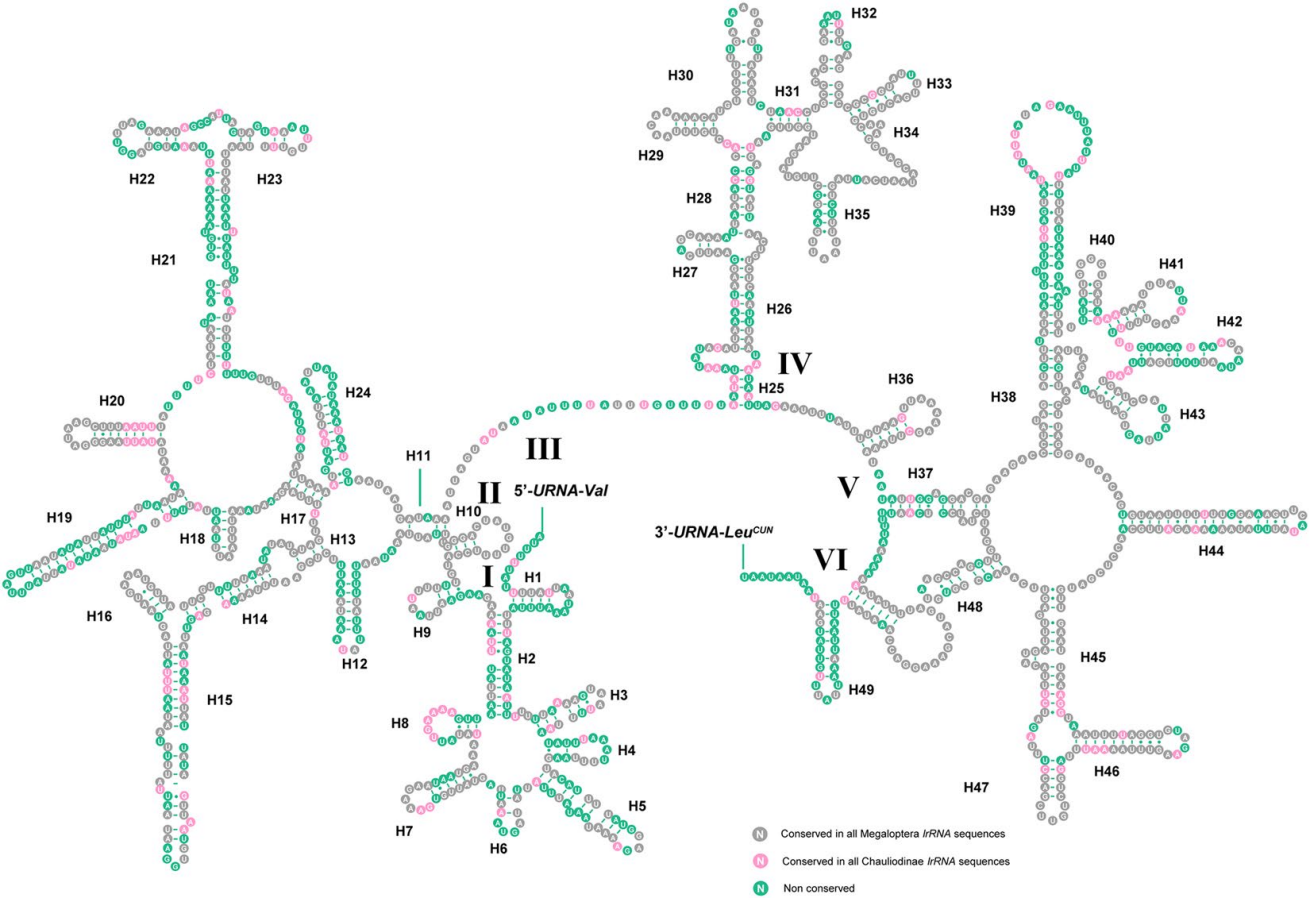
Predicted secondary structure of the *lrRNA* in the *Archichauliodes deceptor* mt genome. Roman numerals denote the conserved domain structure. Dash (−) indicates Watson-Crick base pairing and dot (∙) indicates G-U base pairing.

**Figure 7:**
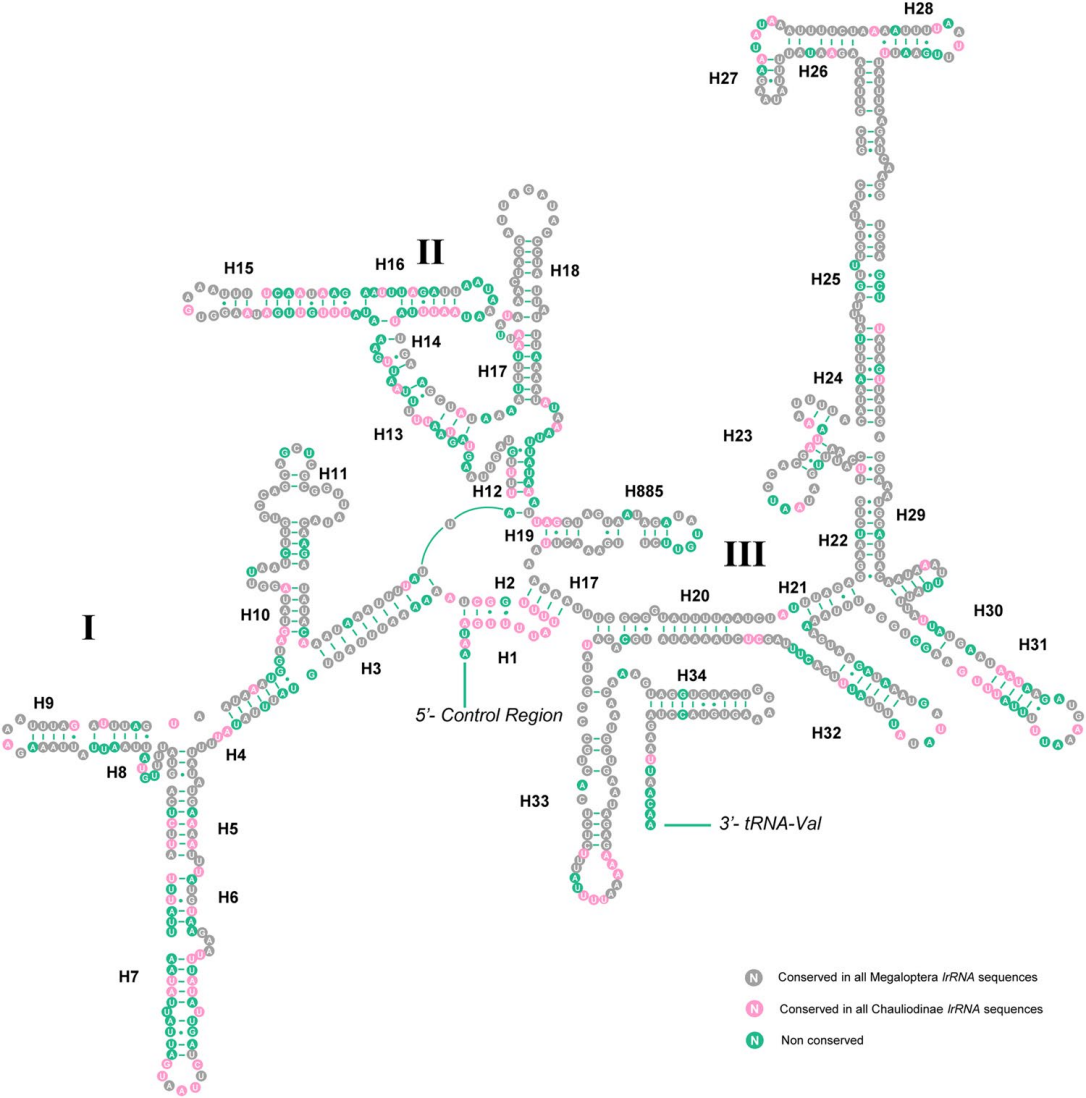
Predicted secondary structure of the *srRNA* in the *Archichauliodes deceptor* mt genome. Roman numerals denote the conserved domain structure. Dash (−) indicates Watson-Crick base pairing and dot (∙) indicates G-U base pairing.

We inferred the secondary structure of *lrRNA* and *srRNA* of *A*. *deceptor* using the published rRNA structure of *Neoneuromus tonkinensis* ^8^ and *Agriosphodrus dohrni* ^9^ as models. There were 49 helices in *lrRNA* in five structural domains (I-II, IV-VI), domain III is absent as in other arthropods (Fig. 6)^10^. The multiple alignments of Chauliodinae and Megaloptera indicate that conserved nucleotides were distributed unevenly throughout the *lrRNA* secondary structure. In addition, most invariable positions were found within domain IV, while the lower conserved positions were in domains I-II. The secondary structure of *srRNA* contains three domains (Fig. 7), and it is less conservative than *lrRNA*. The H7 region within *srRNA* is highly variable and difficult to predict among different insects.

### Nucleotide composition and codon usage

The nucleotide composition of mt genomes of *A*. *deceptor* and *P*. *biconicus* is clearly biased towards A/T nucleotides (*A*. *deceptor*: A = 39.5%, T = 38.0%, C = 13.9%, G = 8.6%; *P*. *biconicus*: A = 38.0%, T = 39.0%, C = 13.7%, G = 9.3%) (Tables S2–S3). The A + T content is much higher than G + C content in all mt genes of *A*. *deceptor* and *P*. *biconicus*, respectively, i.e. PCGs (75.8%, 76.0%), tRNAs (78.5%, 76.5%), rRNAs (81.0%, 81.2%) and the control region (86.8%). The AT-Skew and GC-Skew in the mt genome of *A*. *deceptor* are 0.019 and −0.236, while in *P*. *biconicus* they are −0.013 and −0.191.

The codon usage of all PCGs in the mt genomes of *A*. *deceptor* and *P*. *biconicus* is similar to that in other invertebrates (Tables S4–S5). We found that NNU was the most frequently used codon, while NNA was only used in Leu (UUR), Met (AUN), Gln (CAN), Lys (AAN), Glu (GAN) and Gly (GGN). NNG and NNC are less used codons. In addition, A and T bias is reflected in the codon usage, such that the A + T rich codons, i.e. TTT-Phe, TTA-Leu, ATT-Ile, ATA-Met, TAT-Tyr, AAT-Asn and AAA-Lys, are more frequently used than the G + C rich codons.

### Phylogenetic analysis and divergence time estimation

The ML and BI analyses based on the final dataset of 13,247 nucleotide sites generated the phylogenic trees with same topologies and high nodal supports (Fig. 7). Corydalinae and Chauliodinae were both monophyletic and together formed a monophyletic Corydalidae, which is the sister group of Sialidae. In Chauliodinae, *Dysmicohermes* was assigned the sister group of the remaining genera of fishflies. *Protochauliodes* was recovered as the sister group of *Archichauliodes* + *Neochauliodes*.

**Figure 8:**
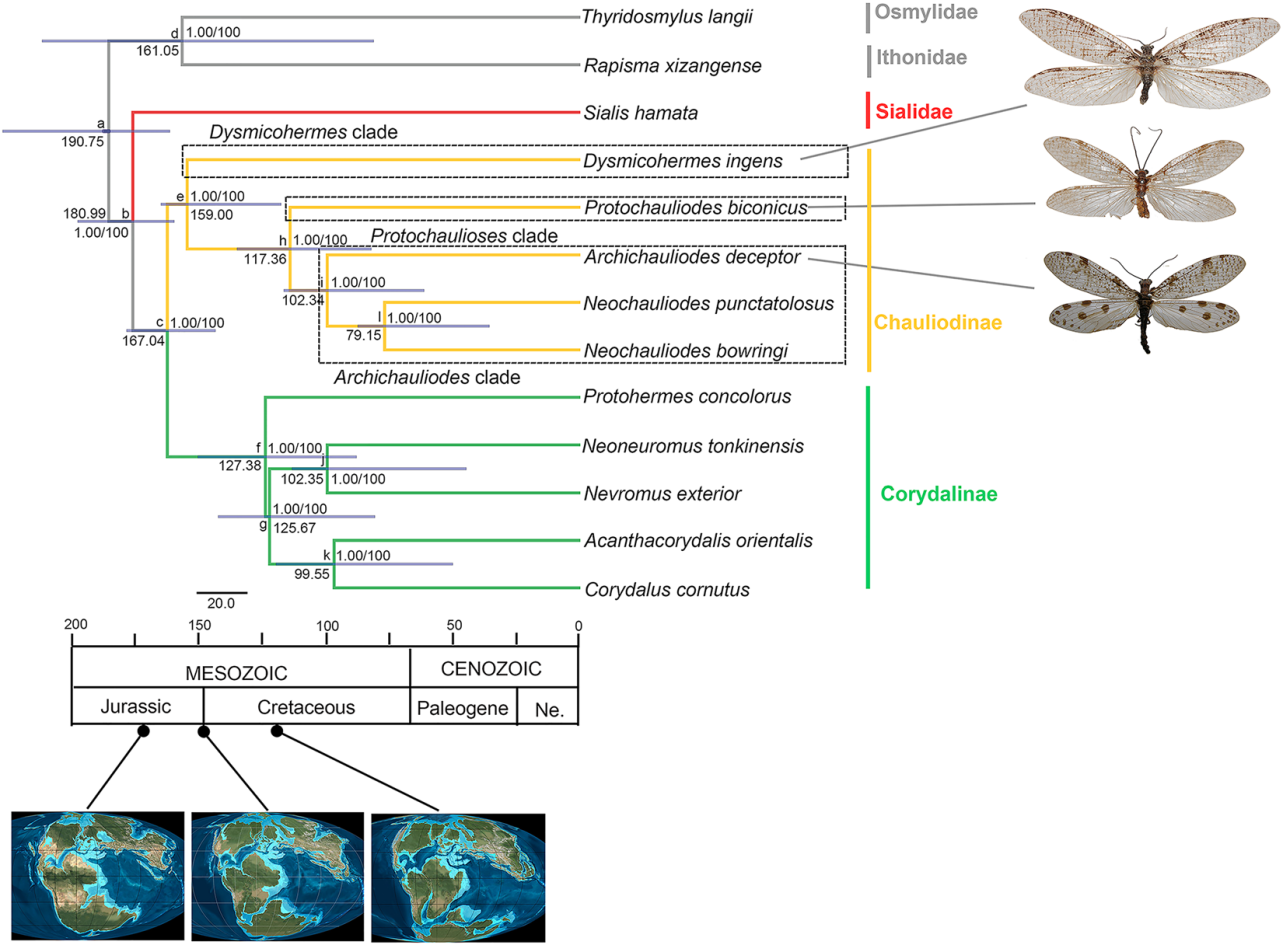
Chronogram showing relationships among major subgroups of Chauliodinae inferred from mt genome sequences. Numbers at the nodes are Bayesian posterior probabilities (left) and ML bootstrap values (right). Nodes on the chronogram represent means of the probability distributions for node ages, with time intervals for 95% probability of actual age represented as blue bars (Table 3). Scale units are in millions of years, and numbers on nodes represent the estimated age for that divergence. Geologic periods calibrated to a time scale in Myr are placed at bottom, with rough images of palaeogeographical composition (acquired from Global Paleogeography and Tectonics in Deep Time © 2016 Colorado Plateau Geosystems Inc.).

The estimation of divergence times among the sampled Megaloptera taxa showed a Mesozoic diversification for the extant families and subfamilies of Megaloptera, as well as for the genera of Corydalidae (Fig. 8, Table 3). In Chauliodinae, the divergence between *Dysmicohermes* and the other genera of fishflies was dated to be in the Late Jurassic ca. 159 MA (95% HPD 121.01–169.64 MA), which is slightly earlier than the estimate in Wang *et al*.^11^ (ca. 140 MA/95%HPD 67.96–234.89 MA); however, in consideration of the 95% credibility interval, the current estimate falls within the range of the previous study^11^. *Protochauliodes* was estimated to have diverged from *Archichauliodes* + *Neochauliodes* in the Early Cretaceous, ca. 117 MA. Divergence between *Archichauliodes* and *Neochauliodes* was also dated in the Early Cretaceous, ca. 102 MA. In Corydalinae, the divergence times among the five dobsonfly genera were estimated to be in the Cretaceous, which corresponds to results in Winterton *et al*.^12^ and Wang *et al*.^11^.

**Table 3:** The maximum clade credibility tree with median node heights and the 95% high posterior density (HPD) interval on each divergence.

Node	mean	Inferior 95%	Superior 95%
a	190.75	166.02	233.65
b	180.99	164.24	203.28
c	167.04	147.55	183.53
d	161.05	83.56	217.69
e	159.00	121.01	169.64
f	127.38	90.45	154.71
g	125.67	83.05	146.47
h	117.36	84.50	138.87
i	102.34	63.16	119.91
j	102.35	46.11	116.64
k	99.55	51.56	123.08
l	79.15	36.73	90.03

Time-scale units are in millions of years.

## Discussion

### Phylogenetic considerations

The species of fishflies investigated here from *Archichauliodes* and *Protochauliodes* are significant for inferring the phylogeny of Chauliodinae, since they represent two major subgroups of fishflies, i.e. the *Archichauliodes* clade and the *Protochauliodes* clade, as proposed by a recent morphology-based phylogeny of fishflies^4^. Aside from these two clades, the remaining major subgroup of Chauliodinae is the *Dysmicohermes* clade. Our phylogenetic analysis is the first to use molecular data to test the relationships of these three major fishfly subgroups. Our results are generally consistent with the previous morphology-based phylogeny, in which the *Dysmicohermes* clade was the sister group of the *Archichauliodes* clade + the *Protochauliodes* clade, although we could not corroborate the monophyly of each clade due to lack of many genera.

*Dysmicohermes*, together with its sister genus *Orohermes*, are basal fishflies and possess a number of plesiomorphic morphological characters, such as the moderately developed male gonocoxites and gonostyli 9, and the feebly produced spiracles on the larval abdominal segment 8^2, 4^. Furthermore, the wing venation and larval morphology of *Dysmicohermes* and *Orohermes* largely resemble that in *Jurochauliodes* which is one of the most ancestral fishfly genera currently known from the Middle Jurassic of China. Thus, the *Dysmicohermes* clade should be considered as the basal most subgroup among extant Chauliodinae on the basis of morphological and molecular evidence. The divergence time estimation indicated that the evolutionary history of this subgroup is considerably long and dates to the earliest Late Jurassic (ca. 159 MA).

The *Protochauliodes* clade currently includes five genera, four of which possess a distinct wing character (i.e., anterior branch of 2 A partially fused with stem of 1 A) which supports the autapomorphic nature of these genera^4^. Furthermore, additional genital characters indicate autapomorphies of the *Protochauliodes* clade^2^. This clade was estimated by our analysis to have diverged with the *Archichauliodes* clade during the Early Cretaceous. However, Liu *et al*. (2012) postulated that these two clades might have diverged during the Early Jurassic. In fact, this hypothesis was proposed based on the inclusion of a fossil fishfly genus in the *Protochauliodes* clade, namely *Eochauliodes* from the Middle Jurassic of China, which accordingly prolonged the interpretation on the evolutionary history of this clade. Nevertheless, the evidence supporting the assignment of *Eochauliodes* in the *Protochauliodes* clade is weak, since it is a homoplasious wing venation character, i.e., bifurcated anterior branch of forewing Rs. Future studies may reveal that this Middle Jurassic fossil genus is distantly related and diverged much earlier than the extant *Protochauliodes* clade and the *Archichauliodes* clade.

### Biogeographic considerations

Previous study on the phylogeny and historical biogeography of Chauliodinae suggested a Pangaean origin and a global distribution of the subfamily before the Middle Jurassic, while the earliest diversification of fishflies might have occurred before the initial split of Pangaea^4^. Moreover, Liu *et al*. (2012) considered that the divergence among the three major fishfly clades might have taken place when Pangaea was not yet separated. However, our estimate for divergence between the *Dysmicohermes* clade and the *Archichauliodes* clade + the *Protochauliodes* clade is slightly after the initial breakup of Pangaea, which led to the formation of Laurasia and Gondwana during an interval of 180–160 MA^13^. Since the *Archichauliodes* and *Protochauliodes* clades were considered to have originated from Gondwana, the divergence of the *Dysmicohermes* clade, which is endemic to western North America, could be correlated to the geographic vicariance formed by the separation of Laurasia and Gondwana.

The *Archichauliodes* and *Protochauliodes* clades include many austral endemic genera, which were thought to have diverged in connection with the sequential breakup of Gondwana^4^. As mentioned above, the molecule-based result of the divergence time between these two clades is much later than that inferred from the morphological data, with the molecular estimate being ca. 117 MA (95% HPD 84.50–138.87 MA) in the Early Cretaceous. The sequential breakup of Gondwana continued into the Early Jurassic and Late Cretaceous. By ca. 120 MA, Gondwana had split into several landmasses, e.g. Africa + northern South America, Madagascar + India, and a landmass including Antarctica, Australia, southern South America, etc^13^. In Chauliodinae, both *Archichauliodes* and *Protochauliodes* clades include extant genera distributed in the areas belonging to at least two of the above main Gondwanan landmasses. If the initial divergence between the *Archichauliodes* and *Protochauliodes* clades took place after 120 MA, it would be difficult to infer any correlation of the intergeneric divergence within either of these two major clades to Gondwanan plate drifting. Moreover, the disjunct distribution of the austral endemic genera in the *Archichauliodes* and *Protochauliodes* clades seems to be insufficiently explained since fishflies possess a relatively weak capacity for long-distance dispersal. Therefore, the initial divergence between the *Archichauliodes* and *Protochauliodes* clades might have been much earlier than 120 MA. Based on the range of their divergence time presently estimated (95% HPD 84.50–138.87 MA), it is plausible to assume that these two clades might have diverged in the Early Cretaceous ca. 120–139 MA. Thus, subsequent intergeneric divergences, especially for those austral genera, could have been caused by Gondwanan plate drifting.

## Conclusions

The present study is the first to present a phylogenetic analysis based on mt genomic data to infer relationships among the major subgroups of Chauliodinae. Similar to the previous morphology-based intergeneric phylogeny of fishflies, the present results indicate that the *Dysmicohermes* clade is the sister group of the *Archichauliodes* clade + the *Protochauliodes* clade. However, the timescale we estimated for the divergence among the three major subgroups is much later than that hypothesized from the morphology-based phylogeny^4^, suggesting these major divergences were possibly infected by the sequential breakup of Pangaea during the Late Jurassic and Early Cretaceous. Unfortunately, it is still hard to reveal any clear divergence pattern of the whole subfamily due to lack of many genera, particularly those endemic to certain austral landmasses. Future study should focus on a total-evidence analysis with comprehensive sampling to elucidate the evolutionary history of fishflies.

## Material and Methods

### Specimens and DNA extraction

The specimen of *A*. *deceptor* was collected by S. L. Winterton and J. S. Bartlett at Scrub Rd (27.427°S 152.841°E), Brisbane Forest Pk, SE Queensland, Australia, between December 2007 and January 2008. The specimen of *P*. *biconicus* was collected by H. Karube at Brisbane, Australia, on November 12, 2005. After collection, the samples were initially preserved in 95% ethanol in the field, and transferred to −20 °C for the long-term storage upon arrival at the China Agricultural University (CAU). All samples were examined and identified by Xingyue Liu. The genomic DNA was extracted and purified from the mesothoracic muscle using TIANamp Genomic DNA Kit (TIANGEN).

### PCR amplification and sequencing

The mt genomes of *A*. *deceptor* and *P*. *biconicus* were generated by amplification of overlapping PCR fragments. PCR primers we used included universal and specifically designed primers (Tables S6–S7). All PCRs were performed using NEB Long Taq DNA polymerase (New England Biolabs, Ipswich, MA) under the following amplification conditions: 95 °C for 30 s, 40 cycles of denaturation at 95 °C for 10 s, annealing at 43–55 °C (depending on the primer pair used) for 50 s, elongation at 65 °C for 1 kb/min (depending on the size of amplicon), and the final elongation step at 65 °C for 10 min. The quality of PCR products was assessed through electrophoresis in a 1% agarose gel and staining with Gold View.

All PCR products were sequenced in both directions using the BigDye Terminator Sequencing Kit (Applied Bio Systems) and the ABI 3730XL Genetic Analyzer (PE Applied Biosystems, San Francisco, California USA) with two vector-specific primers and internal primers for primer walking.

### Bioinformatic analysis

The complete mt genomes of *A*. *deceptor* and *P*. *biconicus* are deposited in GenBank with accession numbers KU925864 and KY230493, respectively. Sequence assembly was done using ContigExpress. As for the sequence analysis, the tRNAs were identified by tRNAscan-SE Search Server v. 1.21^14^, while for those tRNAs which could not be detected by this program we compared them with the corresponding tRNAs gene sequence of *Neochauliodes punctatolosus* Liu & Yang^5^ to determine the position and sequence. The annotations of PCGs and rRNA genes were verified by hand alignment with closely-related species of Chauliodinae. The control region was identified afterwards by the boundary of the rRNA genes and compared with other insect mt genomes. Nucleotide substitution rates, base composition and codon usage were analyzed with MEGA 5.0^15^ The GC and AT skews were measured using the following formulae: AT-skew = (A − T)/(A + T) and GC-skew = (G − C)/(G + C)^16^.

### Phylogenetic analysis

Nine species of Megaloptera with determined mt genomes were included in the ingroup taxa and the outgroup taxa comprised two species of Neuroptera, namely *Thyridosmylus langii* (McLachlan) (Neuroptera: Osmylidae) and *Rapisma xizangense* (Neuroptera: Ithonidae) (Table 4). Sequences of 13 PCGs and two rRNAs were used in the present phylogenetic analysis. The PCGs were aligned based on the amino acid alignment using ClustalW in MEGA 5.0^15^. RNA alignment was conducted by G-blocks Server (http://molevol.cmima.csic.es/castresana/Gblocks_server.html). Individual genes were concatenated by SequenceMatrix v1.7.8^17^. We performed maximum likelihood (ML) and Bayesian inference (BI) using the best-fit partitioning schemes recommended by PartitionFinder^18^. For the ML analysis, we ran 1,000 bootstrap replicates and used the rapid bootstrap feature (random seed value 12345) in RAxML^19^. MrBayes 3.2.2^20^ was used to conduct the BI analysis with the GTR + I + G model as the optimal model selected by PartitionFinder. Two simultaneous runs of 2 million generations were conducted for the dataset, the tree samples were outputted every 1,000 generations with a burnin of 25%.

**Table 4:** Taxa used in the phylogenetic analysis.

Order	Family/Subfamily	Species	Accession number
Megaloptera	Corydalidae/Chauliodinae	*Archichauliodes deceptor*	KU925864
	Corydalidae/Chauliodinae	*Neochauliodes punctatolosus*	NC_018772
	Corydalidae/Chauliodinae	*Neochauliodes bowringi*	NC_023444
	Corydalidae/Chauliodinae	*Dysmicohermes ingens*	NC_024657
	Corydalidae/Corydalinae	*Protohermes concolorus*	NC_011524
	Corydalidae/Corydalinae	*Neoneuromus tonkinensis*	NC_027852
	Corydalidae/Corydalinae	*Nevromus exterior*	NC_027851
	Corydalidae/Corydalinae	*Acanthacorydalis orientalis*	NC_023462
	Corydalidae/Corydalinae	*Corydalus cornutus*	NC_011276
	Sialidae	*Sialis hamata*	NC_013256
Neuroptera	Osmylidae	*Thyridosmylus langii*	NC_021415
	Ithonidae	*Rapisma xizangense*	KF626447

### Divergence time estimation

Estimation of divergence times was conducted with all mt genome data using BEAST version 1.5.3^21^. The taxa and data partitioning we used were consistent with the previous phylogenetic analysis using the GTR + I + G model, estimated base frequencies and Yule process of speciation. Minimum node constrains were assigned a normal prior distribution with standard deviations equal to 12 Ma.

Due to the difficulty of fossilization in habitats associated with fast-flowing water^22^, there are scarce fossil records of Megaloptera. We set two fossil calibrations in our analysis (1) the mean age of Corydalidae + Sialidae was set at 185 MA with the 95% credibility interval around the mean spanning the period from 204.7 to 165.3 MA, reflecting the minimum age of these two families, which is based on oldest known fossil of Sialidae (*Dobbertinia reticulata* Handlirsch) from the Lower Jurassic of Dobbertin, Germany (~185 MA)^23^; (2) the mean age of Chauliodinae + Corydalinae was set at 165 MA with the 95% credibility interval around the mean spanning the period from 184.7 to 145.3 Ma based on the fossil evidence of an adult fishfly (*Jurochauliodes ponomarenkoi* Wang & Zhang) from the Middle Jurassic of Inner Mongolia, China (~165 MA) reported in Liu *et al*.^4^. Two independent MCMC analyses were run for 5 million generations under the uncorrelated lognormal relaxed clock model and sampled every 1000 generations. We combined tree files of both runs using LogCombiner 1.5.3, with the first 25% of the generations from each run discarded as burnin. Finally, we used TreeAnnotator 1.5.3^21^ to calculate divergence time from a combined tree file. The phylogenic tree was viewed and edited using FigTree 1.3.1^24^.

## Electronic supplementary material

Supplementary Information

## References

[1] Yang, D. & Liu, X. Y. *Fauna Sinica Insecta Vol*.*51 Megaloptera* (Science Press, 2010).

[2] Liu XY, Lü YN, Aspöck H, Yang D, Aspöck U (2016). Homology of the genital sclerites of Megaloptera (Insecta: Neuropterida) and their phylogenetic relevance. Syst. Entomol..

[3] New TR, Theischinger G (1993). Megaloptera (Alderflies, Dobsonflies). Handbuch der Zoologie.

[4] Liu XY, Wang YJ, Shih CK, Ren D, Yang D (2012). Early evolutionary and historical biogeography of fishflies (Megaloptera: Chauliodinae): implications from a phylogeny combining fossil and extant taxa. PLoS ONE.

[5] Wang YY, Liu XY, Winterton SL, Yang D (2012). The first mitochondrial genome for the fishfly subfamily Chauliodinae and implications for the higher phylogeny of Megaloptera. PLoS ONE.

[6] Clary D, Wolstenholme DR (1985). The mitochondrial DNA molecule of *Drosophila yakuba*: Nucleotide sequence, gene organization, and genetic code. J. Mol. Evol..

[7] Beckenbach AT, Stewart JB (2009). Insect mitochondrial genomics 3: the complete mitochondrial genome sequences of representatives from two neuropteroid orders: a dobsonfly (order Megaloptera) and a giant lacewing and an owlfly (order Neuroptera). Genome.

[8] Jiang YL (2015). Complete mitochondrial genomes of two Oriental dobsonflies, *Neoneuromus tonkinensis* (van der Weele) and *Nevromus exterior* (Navás) (Megaloptera: Corydalidae), and phylogenetic implications of Corydalinae. Zootaxa.

[9] Li H, Gao JY, Liu HY, Liang AP, Cai WZ (2011). The architecture and complete sequence of mitochondrial genome of an assassin bug *Agriosphodrus dohrni* (Hemiptera: Reduviidae). Int. J. Biol. Sci..

[10] Dowton M, Castro LR, Austin AD (2002). Mitochondrial gene rearrangements as phylogenetic characters in the invertebrates: the examination of genome ‘morphology’. Invertebr Syst..

[11] Wang YY (2017). Mitochondrial phylogenomics illuminates the evolutionary history of Neuropterida. Cladistics..

[12] Winterton SL, Hardy NB, Wiegmann BM (2010). On wings of lace: phylogeny and Bayesian divergence time estimates of Neuropterida (Insecta) based on morphological and molecular data. Syst. Entomol.

[13] Sanmartín L, Ronquist F (2004). Southern hemisphere biogeography inferred by event-based models: Plant versus animal patterns. Syst. Biol..

[14] Lowe TM, Eddy SR (1997). tRNAscan–SE: a program for improved detection of transfer RNA genes in genomic sequence. Nucleic Acids Res.

[15] Tamura K (2011). MEGA5: Molecular evolutionary genetics analysis using maximum likelihood, evolutionary distance, and maximum parsimony methods. Mol. Biol. Evol..

[16] Perna NT, Kocher TD (1995). Patterns of nucleotide composition at fourfold degenerate sites of animal mitochondrial genomes. J. Mol. Evol..

[17] Vaidya G, Lohman DJ, Meier R (2010). SequenceMatrix: concatenation software for the fast assembly of multi-gene datasets with character set and codon information. Cladistics..

[18] Lanfear R, Calcott B, Ho SYW, Guindon S (2012). PartitionFinder: Combined selection of partitioning schemes and substitution models for phylogenetic analysis. Mol. Biol. Evol..

[19] Stamatakis A, Hoover P, Rougemont J (2008). A rapid bootstrap algorithm for the RAxML Web servers. Syst. Biol..

[20] Ronquist F, Huelsenbeck JP (2003). MrBayes 3: Bayesian phylogenetic inference under mixed models. Bioinformatics.

[21] Drummond A, Rambaut A (2007). BEAST: Bayesian evolutionary analysis by sampling trees. BMC Evol. Biol..

[22] Ponomarenko A (1976). Corydalidae (Megaloptera) from Cretaceous deposits of northern Asia. Entomol. Obozr..

[23] Ansorge J (2001). *Dobbertinia reticulata* Handlirsch 1920 from the Lower Jurassic of Dobbertin (Mecklenburg/Germany) – the oldest representative of Sialidae (Megaloptera). N. Jb. Geol. Palaönt. Mh.

[24] Rambaut, A. FigTree version 1.3. 1. *Computer program distributed by the author* Available: http://tree bio ed ac uk/software/figtree/ (Accessed 2011 Jan 4).

[25] Grant JR, Stothard P (2008). The CGView Server: a comparative genomics tool for circular genomes. Nucleic Acids Res.

