# Supplementary material for: Mitochondrial genomes of two Australian fishflies with an evolutionary timescale of Chauliodinae
Source: Sci Rep. 2017 Jun 30;7:4481. doi: 10.1038/s41598-017-04799-y (PMC5493631; doi:10.1038/s41598-017-04799-y)
Supplement: Supplementary file 1 — Supplementary Information [file 41598_2017_4799_MOESM1_ESM.pdf]

Mitochondrial genomes of two Australian fishflies with an evolutionary timescale of Chauliodinae

Fan Yang, Yunlan Jiang, Ding Yang, Xingyue Liu*

Department of Entomology, China Agricultural University, Beijing 100193, China.

*Correspondence: Xingyue Liu, Department of Entomology, China Agricultural University, Beijing 100193, China. Email: xingyue_liu@yahoo.com

Table S1. The Size of PCGs, tRNAs, *lrRNA*, *srRNA*, and CR, respectively, among sequenced Megeloptera mt genomes

Species	PCGs	tRNAs	*lrRNA*	*srRNA*	CR
Neochauliodes punctatolosus	11166.00	1449.00	1318.00	789.00	1006.00
Neochauliodes bowringi	11165.00	1460.00	1317.00	787.00	1328.00
Neochauliodes fraternus	11170.00	1453.00	1309.00	786.00	1015.00
Neochauliodes rotundatus	11187.00	1459.00	1314.00	790.00	1045.00
Dysmicohermes ingens	11196.00	1452.00	1320.00	792.00	1006.00
Archichauliodes deceptor	11175.00	1446.00	1318.00	790.00	1050.00
Sialis hamata	11192.00	1515.00	1321.00	784.00	814.00
Protohermes concolorus	11184.00	1479.00	1312.00	774.00	1131.00
Corydalus cornutus	11175.00	1504.00	1311.00	786.00	967.00
Neoneuromus tonkinensis	11175.00	1448.00	1313.00	789.00	1048.00
Nevromus exterior	11154.00	1440.00	1309.00	788.00	1053.00
Acanthacorydalis orientalis	11171.00	1453.00	1306.00	786.00	1006.00
Avg.	11175.83	1463.17	1314.00	786.75	1039.08

Supplementary Table S2. Nucleotide composition of the *Archichauliodes deceptor* mt genome.

Feature	A%	T%	C%	G%	A+T%	AT-skew	GC-skew
Whole genome	39.5	38.0	13.9	8.6	77.5	0.019	-0.236
protein–coding genes	38.4	37.4	14.9	9.3	75.8	0.01	-.0231
First codon position	41.5	36	12.0	10.7	77.5	-0.161	-0.057
Second codon position	33.0	35	19.6	12.2	68.0	-0.124	-0.233

Third codon position	40.7	41	13.2	4.9	81.7	-0.145	-0.459
protein–coding genes J-strand	33.3	41.2	14.8	10.7	74.5	-0.106	-0.161
First codon position	39.9	39	10.9	9.9	78.9	0.011	-0.048
Second codon position	28.4	38	18.3	14.8	66.4	-0.145	-0.106
Third codon position	31.6	46	15.1	7.5	77.6	-0.186	-0.336
protein–coding genes N-strand	46.6	31.3	15.1	7.0	77.9	0.196	-0.367
First codon position	45.9	25	20.0	9.4	70.9	0.295	-0.361
Second codon position	45.5	36	14.0	4.7	81.5	0.117	-0.497
Third codon position	48.3	34	11.3	6.8	82.3	0.174	-0.249
tRNA genes	39.8	38.7	12.2	9.4	78.5	0.014	-0.130
tRNA genes J-strand	40.2	37.8	10.8	11.3	78.0	0.031	0.023
tRNA genes N-strand	39.1	40.2	14.6	6.1	79.3	-0.014	-0.411
rRNA genes	42.7	38.3	12.7	6.3	81.0	0.054	-0.337
Control region	44.6	42.2	8.6	4.6	86.8	0.028	-0.303

Supplementary Table S3. Nucleotide composition of the *Protochauliodes biconicus* mt genome.

Feature	A%	T%	C%	G%	A+T%	AT-skew	GC-skew
Whole genome	38.0	39.0	13.7	9.3	77.0	-0.013	-0.191
protein–coding genes	31.9	44.1	11.8	12.1	76.0	-0.161	0.013
First codon position	34.1	42	10.0	14.3	76.1	-0.104	0.177
Second codon position	25.8	44	15.7	14.4	69.8	-0.261	-0.043
Third codon position	35.8	47	9.8	7.7	82.8	-0.135	-0.12
protein–coding genes J-strand	31.7	42.8	14.4	11.1	74.5	-0.149	-0.129
First codon position	31.1	37	15.1	16.8	68.1	-0.087	0.053
Second codon position	24.7	44	18.5	12.4	68.7	-0.281	-0.197
Third codon position	39.3	47	9.5	4.0	86.5	-0.089	-0.407
protein–coding genes N-strand	32.8	45.9	7.7	13.7	78.7	-0.166	0.076
First codon position	39.5	41	4.8	14.2	80.5	-0.019	0.495
Second codon position	23.5	49	11.7	16.2	72.5	-0.352	0.161
Third codon position	35.3	48	6.4	10.6	83.3	-0.152	0.247
tRNA genes	39.4	37.1	9.7	13.7	76.5	0.03	0.171
tRNA genes J-strand	39.7	38.0	10.5	11.8	77.7	0.022	0.058
tRNA genes N-strand	39.3	36.4	7.9	16.3	75.7	0.038	0.347
rRNA genes	38.9	42.3	6.4	12.4	81.2	-0.042	0.319

Supplementary Table S4. Codon usage of the *Archichauliodes deceptor* mt genome. N, total number in all proteins; N+, total number in J-strand; N-, total number in N-strand; RSCU, relative synonymous codon usage. Values in bold type stand for the most commonly used

codon for the amino acid. Underlined codons stand for the cognate codon of tRNA for each amino acid.

Amino acid	Codon	N	RSCU	N+	RSCU	N-	RSCU
Phe (F)	**UUU**	**219**	**1.55**	**182**	**1.58**	**36**	**1.33**
	UUC	64	0.45	49	0.42	18	0.67
Leu^{UUR} (L)	**UUA**	**209**	**3.63**	**146**	**3.6**	**80**	**2.3**
	UUG	20	0.35	23	0.57	16	0.46
Leu^{CUN} (L)	**CUU**	**57**	**0.99**	**44**	**1.09**	**33**	**0.95**
	CUC	9	0.16	6	0.15	12	0.34
	CUA	44	0.77	21	0.52	59	1.69
	CUG	6	0.1	3	0.07	9	0.26
Ile (I)	**AUU**	**299**	**1.76**	**214**	**1.75**	**86**	**1.52**
	AUC	40	0.24	30	0.25	27	0.48
Met (M)	**AUA**	**221**	**1.75**	**86**	**1.52**	**111**	**1.91**
	AUG	32	0.25	27	0.48	5	0.09
Val(V)	GUU	44	2.12	26	2.04	5	0.95
	GUC	2	0.1	2	0.16	4	0.76
	**GUA**	**36**	**1.73**	**22**	**1.73**	**11**	**2.1**
	GUG	1	0.05	1	0.08	1	0.19
Ser (S)	**UCU**	**98**	**1.78**	**59**	**1.51**	**18**	**3.2**
	UCC	59	1.07	32	0.82	5	0.89
	UCA	101	1.83	69	1.77	17	3.02
	UCG	11	0.2	8	0.21	0	0
Pro (P)	**CCU**	**56**	**1.52**	**31**	**1.59**	**32**	**1.8**
	CCC	32	0.87	17	0.87	13	0.73
	**CCA**	**56**	**1.52**	**27**	**1.38**	**26**	**1.46**
	CCG	3	0.08	3	0.15	0	0
Thr (T)	**ACU**	**93**	**1.75**	**58**	**1.92**	**21**	**1.4**
	ACC	35	0.66	24	0.79	16	1.07
	ACA	70	1.31	30	0.99	23	1.53
	ACG	15	0.28	9	0.3	0	0
Ala (A)	**GCU**	**48**	**1.96**	**28**	**2.11**	**10**	**1.33**
	GCC	14	0.57	9	0.68	5	0.67
	GCA	36	1.47	15	1.13	15	2
	GCG	0	0	1	0.08	0	0
Tyr (Y)	**UAU**	**173**	**1.5**	**130**	**1.51**	**31**	**1.27**
	UAC	57	0.5	42	0.49	18	0.73
Stop (*)	UAA	170	1.9	42	1.56	75	1.61

	UAG	9	0.1	12	0.44	18	0.39
His (H)	**CAU**	**51**	**1.44**	**29**	**1.35**	**31**	**1.51**
	CAC	20	0.56	14	0.65	10	0.49
Gln (Q)	**CAA**	**73**	**1.95**	**33**	**1.94**	**36**	**1.57**
	CAG	2	0.05	1	0.06	10	0.43
Asn (N)	**AAU**	**250**	**1.54**	**150**	**1.43**	**133**	**1.66**
	AAC	74	0.46	60	0.57	27	0.34
Lys (K)	**AAA**	**222**	**1.88**	**69**	**1.66**	**174**	**1.71**
	AAG	14	0.12	14	0.34	29	0.29
Asp (D)	**GAU**	**47**	**1.54**	**23**	**1.53**	**16**	**1.6**
	GAC	14	0.46	7	0.47	4	0.4
Glu (E)	**GAA**	**46**	**2**	**24**	**2**	**34**	**1.74**
	GAG	0	0	0	0	5	0.26
Cys (C)	**UGU**	**22**	**1**	**23**	**1.07**	**6**	**2**
	**UGC**	**22**	**1**	**20**	**0.93**	**0**	**0**
Trp (W)	**UGA**	**76**	**1.65**	**40**	**1.27**	**15**	**2**
	UGG	16	0.35	23	0.73	0	0
Arg (R)	CGU	10	1.14	8	1.45	9	2.25
	CGC	2	0.23	2	0.36	1	0.25
	**CGA**	**23**	**2.63**	**12**	**2.18**	**6**	**1.5**
	CGG	0	0	0	0	0	0
Ser (S)	AGU	31	0.56	29	0.74	2	0.36
	AGC	45	0.82	32	0.82	0	0
	**AGA**	**54**	**0.98**	**41**	**1.05**	**2**	**0.36**
	AGG	42	0.76	42	1.08	1	0.18
Gly (G)	GGU	25	0.91	14	0.82	11	1.76
	GGC	1	0.04	0	0	2	0.32
	**GGA**	**72**	**2.62**	**48**	**2.82**	**8**	**1.28**
	GGG	12	0.44	6	0.35	4	0.64

Supplementary Table S5. Codon usage of the *Protochauliodes biconicus* mt genome. N, total number in all proteins; N+, total number in J-strand; N-, total number in N-strand; RSCU, relative synonymous codon usage. Values in bold type stand for the most commonly used codon for the amino acid. Underlined codons stand for the cognate codon of tRNA for each amino acid.

Amino acid	Codon	N	RSCU	N+	RSCU	N-	RSCU
Phe (F)	**UUU**	**386**	**1.77**	**203**	**1.71**	**120**	**1.68**
	UUC	67	0.23	34	0.29	23	0.32

Leu^{UUR} (L)	**UUA**	**320**	**4.35**	**205**	**3.59**	**108**	**4.53**
	UUG	56	0.4	13	0.23	28	1.17
Leu^{CUN} (L)	**CUU**	**62**	**0.59**	**68**	**1.19**	**4**	**0.17**
	CUC	13	0.06	13	0.23	1	0.04
	CUA	35	0.57	32	0.56	2	0.08
	CUG	13	0.02	12	0.21	0	0
Ile (I)	**AUU**	**307**	**1.85**	**197**	**1.75**	**109**	**1.86**
	AUC	38	0.15	28	0.25	8	0.14
Met (M)	**AUA**	**176**	**1.78**	**107**	**1.83**	**71**	**1.65**
	AUG	32	0.22	10	0.17	15	0.35
Val(V)	**GUU**	**60**	**1.9**	**33**	**1.59**	**22**	**1.87**
	GUC	14	0.1	9	0.43	4	0.34
	GUA	54	1.77	40	1.93	20	1.7
	GUG	2	0.23	1	0.05	1	0.09
Ser (S)	**UCU**	**85**	**4.35**	**57**	**2.35**	**20**	**1.37**
	UCC	33	0.4	14	0.58	4	0.27
	UCA	88	0.59	62	2.56	20	1.37
	UCG	10	0.06	3	0.12	2	0.14
Pro (P)	CCU	54	0.57	57	2.81	10	2.35
	CCC	8	0.02	5	0.25	0	0
	**CCA**	**29**	**1.85**	**16**	**0.79**	**7**	**1.65**
	CCG	4	0.15	3	0.15	0	0
Thr (T)	ACU	71	1.78	62	2.12	11	1.33
	ACC	23	0.22	14	0.48	6	0.73
	**ACA**	**45**	**1.9**	**39**	**1.33**	**10**	**1.21**
	ACG	13	0.1	2	0.07	6	0.73
Ala (A)	GCU	67	1.77	49	2.28	17	2.06
	GCC	16	0.23	14	0.65	1	0.12
	**GCA**	**35**	**4.35**	**23**	**1.07**	**14**	**1.7**
	GCG	1	0.4	0	0	1	0.12
Tyr (Y)	**UAU**	**180**	**0.59**	**77**	**1.38**	**54**	**1.93**
	UAC	50	0.06	35	0.63	2	0.07
Stop (*)	UAA	72	0.57	37	1.42	8	0.76
	UAG	29	0.02	15	0.58	13	1.24
His (H)	**CAU**	**51**	**1.85**	**51**	**1.82**	**7**	**2**
	CAC	4	0.15	5	0.18	0	0
Gln (Q)	**CAA**	**55**	**1.78**	**46**	**1.77**	**14**	**1.87**
	CAG	8	0.22	6	0.23	1	0.13

Asn (N)	**AAU**	**213**	**1.9**	**113**	**1.7**	**80**	**1.88**
	AAC	41	0.1	20	0.3	5	0.12
Lys (K)	**AAA**	**98**	**1.67**	**47**	**1.71**	**35**	**1.59**
	AAG	24	0.33	8	0.29	9	0.41
Asp (D)	**GAU**	**51**	**1.86**	**44**	**1.66**	**12**	**1.85**
	GAC	9	0.14	9	0.34	1	0.15
Glu (E)	**GAA**	**64**	**1.81**	**45**	**1.91**	**16**	**1.78**
	GAG	6	0.19	2	0.09	2	0.22
Cys (C)	**UGU**	**41**	**1.89**	**9**	**1.29**	**20**	**1.67**
	UGC	15	0.11	5	0.71	4	0.33
Trp (W)	**UGA**	**88**	**1.66**	**67**	**1.94**	**18**	**1.2**
	UGG	23	0.34	2	0.06	12	0.8
Arg (R)	CGU	16	1.02	10	1.43	3	1.71
	CGC	1	0.16	0	0	1	0.57
	**CGA**	**14**	**2.59**	**16**	**2.29**	**3**	**1.71**
	CGG	3	0.24	2	0.29	0	0
Ser (S)	AGU	48	0.8	16	0.66	23	1.57
	AGC	23	0.23	8	0.33	6	0.41
	**AGA**	**68**	**2.13**	**31**	**1.28**	**24**	**1.64**
	AGG	43	0	3	0.12	18	1.23
Gly (G)	GGU	40	0.85	24	0.85	13	1.13
	GGC	7	0.14	2	0.07	5	0.43
	**GGA**	**87**	**2.31**	**78**	**2.76**	**19**	**1.65**
	GGG	18	0.69	9	0.32	9	0.78

Supplementary Table S6. Primer sequences of the *Archichauliodes deceptor* mt genome used in this study.

No. fragment *	Primer ID	Nucleotide sequence (5'-3')	Reference
1	F17(SPB-14197)	GTAAAYCTACTTTGTTACGACTT	1
	R17(SPB-14745)	GTGCCAGCAAYCGCGGTTATAC	Present study
2	F187-36F	TAAATAATAGGGTATCTAATCCTAG	Present study
	F187-36R	TAAGAGTTCGTAAATTAAGTTGATT	1
3	F20(SPB-586)	CCATTCCATTTYTGATTTCC	1
	R20(SPB-1738)	TTTATTCGTGGAAATGCTATGTC	Present study
4	F187-31F	TAGGACAACCTGGATCATTAATTG	Present study
	F187-31R	ATACGGTTAGTAATTATGCTTTCTC	1
5	F02(SPA-3399)	TCTATTGGTCAATGGTACTG	1
	R02(SPA-4061)	GAAAATAAATTTGTTATCATTTTCA	1
6	F03(SPA-3790)	CATTAAGTGACTGAAAGCAAGTA	1
	R03(SPA-4552)	ATGACCTGCAATTATATTAGC	Present study
7	F187-33F	CCTCTATGATTAAGCTTTATAATTT	Present study

	F187-33R	CTAATTCGAATGAAGGAGATAAACT	1
8	F05(SPA-4792)	GTAGATGCAAGCCCTTGACC	1
	R05(SPA-5731)	ATTGGATCAAATCCACATTC	1
9	TF5470	GCAGCTGCYTGATAYTGRCA	1
	TR6384	TATATTTAGAGYATRAYAYTGAAG	Present study
10	F187-34F	GTTGTTCAAGATAAAGCTGCTAACT	Present study
	F187-34R	GAAGAGAATTAGGAGGAGGTCAACG	1
11	TF6400	TAACATCTTCAATRTYATRCTCT	1
	TR7211	TTAAGGCTTTAYTATTTATRTGYGC	1
12	F08(SPA-7077)	TTAAATCCTTTGAGTAAAATCC	1
	R08(SPA-7793)	TTAGGTTGAGATAAAATCC	1
13	TF-J7572	AAAGGGAATTTGAGCTCTTTTWGT	1
	TR-N8487	TATCAGSTAATATRGCWGCTCC	1
14	TF-J7806	GAMACAARACCTAACCCATCYCA	1
	TR-N8727	AAATCTTTRATTGCTTATTCWTC	1
15	TF-J8641	CCAGAAGAACATAANCCRTG	1
	TR-N10608	CCAAGTARTGAWCCAAARTTTCA	1
16	TF-J8941	GAAACAGGAGCCTCAACACATGWGC	1
	TR-N10608	CCAAGTARTGAWCCAAARTTTCA	Present study
17	F187-35F	TTTTCCAACAATAAATTCAACTATT	Present study
	F187-35R	CATTTGCATGTAAGGTTCGTAAAAG	1
18	F23(SPC-10621)	CTCATACTGATGAAATTTTGGTTC	1
	R23(SPC-11526)	TTCTACTGGTCGTGCTCCAATTCA	Present study
19	F12(SPB-11335)	CATATTCAACCAGAATGATA	Present study
	R12(SPB-12067)	AATCGTTCTCCATTTGATTTTGC	1
20	F13(SPB-11876)	CGAGGTAAAGTACCACGTACTCA	1
	R13(SPB-12595)	GTTGGATTTCTAACTTTATTRGARCG	1
21	F14(SPB-12261)	TACCTCATAAGAAATAGTTTGAGC	1
	R14(SPB-13000)	TTACCTTAGGGATAACAGCGTAA	1
22	F15(SPB-12888)	CCGGTCTGAACTCAGATCATGTA	1
	R15(SPB-13889)	ATTTATTGTACCTTTTGTATCAG	1
23	F16(SPB-13342)	CCTTTGCACAGTCAAAATACTGC	1
	R16(SPB-14220)	TTATGCACACATCGCCCGTC	1
24	F187-32F	CCAAAATAGAGGTATATCACTCTTA	1
	F187-32R	GGGGCAAGGTATATTTATATTTATA	1

Supplementary Table S7. Primer sequences of the *Protochauliodes biconicus* mt genome used in this study.

No. fragment*	Primer ID	Nucleotide sequence (5’-3’)	Reference
1	F17(SPB-14197)	GTAAAYCTACTTTGTTACGACTT	1
	R17(SPB-14745)	GTGCCAGCAAYCGCGGTTATAC	1
2	ND2-F	AAGCTMMTGGGTTCATACC	Present study
	ND2-R	TATTTTCAGCTTTGAAGGC	Present study
3	H306-09F	CAGCTTTTATACTAATATACCCTTC	Present study
	H306-09R	GAGCTGTGACAATAACATTATAAAT	Present study
4	COI-F	CAACATTTATTTTGATTTTTTGG	Present study
	COI-R	TCCATTGCACTAATCTGCCATATTA	Present study
5	H306-10F	GATCGTATACCTTTATTTGTTTGAT	Present study
	H306-10R	CATCTATTCCTACTGTAAATATATG	Present study
6	LCO1490	GGTCAACAAATCATAAAGATATTGG	Present study
	HCO2198	TAAACTTCAGGGTGACCAAAAAATCA	Present study
7	F22(SPB-2756)	ACATTTTTTCCTCAACATTT	1
	R22(SPB-3389)	TATTCATATCTTCAATATCATTGATG	1
8	F01(SPA-2756)	ACATTTTTTCCTCAACATTT	1

	R01(SPA-3665)	CCACAAATTTCTGAACACTG	1
9	F02(SPA-3399)	TCTATTGGTCAATGGTACTG	1
	R02(SPA-4061)	GAAAATAAATTTGTTATCATTTTCA	1
10	F03(SPA-3790)	CATTAAGTGACTGAAAGCAAGTA	1
	R03(SPA-4552)	ATGACCTGCAATTATATTAGC	1
11	TF3790	CATTAGATGACTGAAAGCAAGTA	1
	TR4908	CGAGTTAYATCTCGTCATCATTG	1
12	H306-31F	TAGGACAACCTGGATCATTAAATTG	Present study
	H306-31R	ATACGGTTAGTAATTATGCTTTCTC	Present study
13	F05(SPA-4792)	GTAGATGCAAGCCCTTGACC	1
	R05(SPA-5731)	ATTGGATCAAATCCACATTC	1
14	TF5470	GCAGCTGCYTGATAYTGRCA	1
	TR6384	TATATTTAGAGYATRAYAYTGAAG	1
15	F187-34F	GTTGTTCAAGATAAAGCTGCTAACT	Present study
	F187-34R	GAAGAGAATTAGGAGGAGGTCAACG	Present study
16	F06(SPA-5747)	CCATTTGAATGTGGRTTTGATCC	1
	R06(SPA-6384)	AAAATTAAAAGCATAATATTGAAG	1
17	TF6400	TAACATCTTCAATRTYATRCTCT	1
	TR7211	TTAAGGCTTTAYTATTTATRTGYGC	1
18	F08(SPA-7077)	TTAAATCCTTTGAGTAAAATCC	1
	R08(SPA-7793)	TTAGGTTGAGATAAAATCC	1
19	H306-34F	CTACCATAATTTAATATTCATGCAAA	Present study
	H306-34R	TTAGTTTAATTTGTTTACGTCAAGT	Present study
20	TF-J8641	CCAGAAGAACATAANCCRTG	1
	TR-N9153	TGAGGTTATCAACCNGARCG	1
21	TF-J8941	GAAACAGGAGCCTCAACACATGWGC	1
	TR-N10608	CCAAGTARTGAWCCAAARTTTCA	1
22	H306-35F	GAAACAGGAGCCTCAACACATGWGC	Present study
	H306-35R	CCAAGTARTGAWCCAAARTTTCA	Present study
23	F11(SPA-9648)	TCCCAACACACCTTCACAAAC	1
	R11(SPA-11010)	TATCAACAGCAAATCCTCCTCA	1
24	F12(SPB-11335)	CATATTCAACCAGAATGATA	1
	R12(SPB-12067)	AATCGTTCTCCATTTGATTTTGC	1
25	F13(SPB-11876)	CGAGGTAAAGTACCACGTACTCA	1
	R13(SPB-12595)	GTTGGATTTCTAACTTTATTRGARCG	1
26	F14(SPB-12261)	TACCTCATAAGAAATAGTTTGAGC	1
	R14(SPB-13000)	TTACCTTAGGGATAACAGCGTAA	1
27	F15(SPB-12888)	CCGGTCTGAACTCAGATCATGTA	1
	R15(SPB-13889)	ATTTATTGTACCTTTTGTATCAG	1
28	F17(SPB-14197)	GTAAAYCTACTTTGTTACGACTT	1
	R17(SPB-14745)	GTGCCAGCAAYCGCGGTTATAC	1

Reference

1. Simon, C., Buckley, T. R., Frati, F., Stewart, J. B. & Beckenbach, A. T. Incorporating molecular evolution into phylogenetic analysis, and a new compilation of conserved polymerase chain reaction primers for animal mitochondrial DNA. *Annu. Rev. Ecol. Evol. Syst.* **37**, 545–579 (2006).
